# Integrated proteomic and metabolomic profiling reveals novel insights on the inflammation and immune response in HFpEF

**DOI:** 10.1186/s12864-024-10575-w

**Published:** 2024-07-08

**Authors:** Muyashaer Abudurexiti, Refukaiti Abuduhalike, Tuersunjiang Naman, Nuerdun Wupuer, Dongqin Duan, Mayire Keranmu, Ailiman Mahemuti

**Affiliations:** Department of Heart Failure, First Affiliated Hospital of Xinjiang Medical University, Urumqi, 830054 Xinjiang China

**Keywords:** HFpEF, Proteomics, Metabolomics, Inflammation, Immune response

## Abstract

**Background:**

The precise mechanisms leading to the development of heart failure with preserved ejection fraction (HFpEF) remain incompletely defined. In this study, an integrative approach utilizing untargeted proteomics and metabolomics was employed to delineate the altered proteomic and metabolomic profiles in patients with HFpEF compared to healthy controls.

**Materials and methods:**

Data were collected from a prospective cohort consisting of 30 HFpEF participants and 30 healthy controls, matched by gender and age. plasma samples were analyzed by multi-omics platforms. The quantification of plasma proteins and metabolites was performed using data-independent acquisition-based liquid chromatography-tandem mass spectrometry (LC-MS/MS) and ultrahigh-performance liquid chromatography-tandem mass spectrometry (UHPLC-MS/MS), respectively. Additionally, Proteomic and metabolomic results were analyzed separately and integrated using correlation and pathway analysis. This was followed by the execution of Gene Ontology (GO) and Kyoto Encyclopedia of Genes and Genomes (KEGG) pathway enrichment studies to elucidate the biological relevance of the observed results.

**Results:**

A total of 46 significantly differentially expressed proteins (DEPs) and 102 differentially expressed metabolites (DEMs) were identified. Then, GO and KEGG pathway enrichment analyses were performed by DEPs and DEMs. Integrated analysis of proteomics and metabolomics has revealed Tuberculosis and African trypanosomiasis pathways that are significantly enriched and the DEPs and DEMs enriched within them, are associated with inflammation and immune response.

**Conclusions:**

Integrated proteomic and metabolomic analyses revealed distinct inflammatory and immune response pathways in HFpEF, highlighting novel therapeutic avenues.

**Supplementary Information:**

The online version contains supplementary material available at 10.1186/s12864-024-10575-w.

## Introduction

Heart failure with preserved ejection fraction (HFpEF) accounts for approximately half of all heart failure cases and is characterized by diastolic dysfunction and preserved left ventricular ejection fraction [1, 2]. HFpEF is initiated by a variety of metabolic-altering factors such as hypertension, obesity, diabetes, and aging [3]. Despite its significant prevalence and substantial clinical impact, HFpEF continues to be a difficult condition to manage. This is attributed to the absence of universally effective therapeutic options and a comprehensive understanding of its underlying pathophysiology [4, 5]. The pathophysiology of HFpEF involves a complex interplay between comorbidities that impose a systemic proinflammatory state, myocardial stiffness, impaired ventricular-arterial coupling, and microvascular endothelial inflammation [6, 7]. however, the metabolic and molecular alterations that ultimately produce these changes are not well established [8].

Proteomics and metabolomics are ‘omics’ technologies that comprehensively analyze the entire proteome and metabolome within specific biological samples [9]. Unlike genetics, which focuses on the hereditary aspects of diseases, proteomics and metabolomics provide insights into the phenotype and progression of diseases [10]. Integrative analysis that combines proteomic and metabolomic profiling has been shown to provide novel insights into the understanding of disease mechanisms and their development [11]. These ‘omics’ technologies enable the comprehensive profiling of proteins and metabolites within biological samples, providing a snapshot of the physiological state and pathophysiological changes occurring in disease conditions [12, 13].

This study analyzed date-independent acquisition (DIA)--based proteomics and high-resolution mass spectrometry metabolomics profiling of plasma samples from HFpEF patients and healthy control. We aim to quantify and identify differentially expressed proteins and metabolites that may contribute to the pathogenesis of HFpEF. The integration of proteomic and metabolomic data through correlation and pathway analysis, complemented by Gene Ontology (GO) and Kyoto Encyclopedia of Genes and Genomes (KEGG) enrichment analyses, allows for a comprehensive examination of the dysregulated biological pathways in HFpEF. The following sections, we will detail the methodology of omics analyses, present the key findings, particular focus on inflammation and immune response, and discuss the potential implications of data for the clinical management and therapeutic targeting of HFpEF.

## Materials and methods

### Patient recruitment and sample collection

With the approvement by the Ethics Committee of the First Affiliated Hospital of Xinjiang Medical University (IRB- K202403-12), Between March to July 2023, a cohort of 30 newly diagnosed HFpEF patients 30 healthy controls were consecutively assembled at the Heart Failure Department of the First Affiliated Hospital of Xinjiang Medical University. These individuals, newly diagnosed with HFpEF upon admission, before the initiation of any treatment, aged between 18 and 85, provided informed consent, and pertinent participant attributes are detailed in Table S1. All participants had been diagnosed with HFpEF according to established consensus criteria [14–16]. The criteria for inclusion involved encompassed symptoms and signs of exertional dyspnea (New York Heart Association class II or III), heart failure with a left ventricular ejection fraction (LVEF) of ≥ 50%, and at least two of the following conditions: [1] elevated NT-proBNP (N-terminal pro-B-type natriuretic peptide) levels ≥ 125 pg/mL; [2] identification of structural heart abnormalities or diastolic dysfunction via echocardiography, and [3] E/e’ ratio ≥ 9. Excluded from participation were individuals with a medical history encompassing congenital heart defects, LVEF < 40%, heart failure categorized as mid-range EF (40–50%), hypertrophic cardiomyopathy, prior cardiac transplantation, constrictive pericarditis, severe valvular disorders, or infiltrative or restrictive cardiomyopathies. The control group was comprised of patients without overt HF characterized by LVEF ≥ 50% and NT-pro-BP concentrations < 125 ng/L.

Plasma samples were collected from patients who received a new diagnosis of HFpEF upon admission, following overnight fasting to minimize dietary influences. Blood was drawn into vacuum-sealed, EDTA-treated tubes to prevent coagulation. After centrifugation at 3000 rpm for 10 min, the supernatant was carefully collected and stored at -80 °C until further analysis.

### Proteome analysis

Utilizing data-independent acquisition (DIA)-based proteomics, LC-MS/MS analysis was conducted to examine proteins in plasma. The proteins were introduced into a mass spectrometer (Q Exactiv HF-X; Thermo Fisher, Germany) linked to a chromatography platform (EASY-nL 1200 UHPLC system; Thermo Fisher, Germany) with DIA mode selected for data collection. Differentially expressed proteins (DEPs) of significant interest were identified based on a fold-change of ≥ 1.5 or ≤ 0.67 and a t-test *p*-value of < 0.05 [9]. Subsequent mapping of DEPs and pathway analysis was facilitated utilizing the KEGG pathway database. Detailed experimental protocol of proteomics was provided in the Supplementary material.

### Metabolomics analysis

Untargeted metabolomic investigations were carried out using LC-MS/MS analysis. The Vanquish UHPLC system (ThermoFisher, Germany) was coupled with either an Orbitrap Q Exactive HF or an Orbitrap Q Exactive HF-X mass spectrometer (Thermo Fisher, Germany) for metabolite separation and identification. Differential expression of metabolites (DEMs) was assessed based on criteria including Variable Importance in Projection (VIP) > 1.0, Fold Change (FC) > 1.2 or FC < 0.833, and a significance level of P-value < 0.05 [9]. The metabolic pathways enriched in DEM were analyzed using the KEGG database. Detailed experimental protocol of metabolomics was provided in the Supplementary material.

### Correlation analysis between proteomics and metabolomics

Pearson correlation analysis was utilized to investigate the relationships between DEPs and DEMs,

considering *p-value* < 0.05 and correlation coefficient > 0. Subsequently, KEGG pathway enrichment analysis was conducted for the DEPs and DEMs.

## Results

### Proteomic profiling analysis of HFpEF and HC samples

**Fig. 1:**
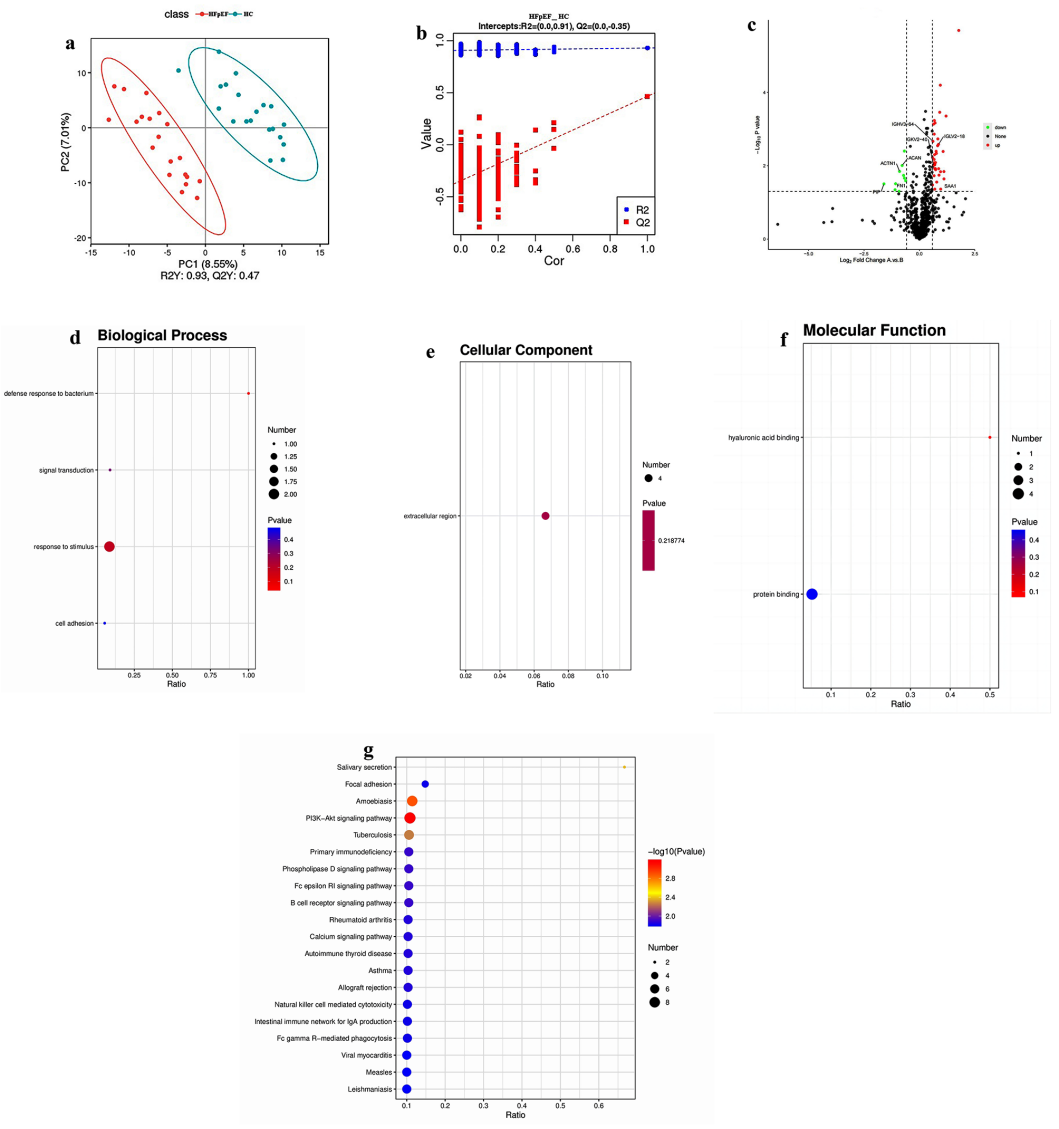
Proteomics analysis in patients with HFpEF compared to HC. PLS-DA score plot (**A**) and Cross-validation plot (**B**). The volcano plot (**C**) showed different proteins in HFpEF compared HC samples. (**D**–**F**) Categories of differentially expressed proteins according to GO. The three major categories of enriched GO functional classification: Biological Process (**D**), Cellular Component (**E**) and Molecular Function (**F**). (**G**) KEGG enrichment analysis of DEPs

Altogether, 6026 peptides and 689 proteins were identified. The PLS-DA analysis used to detect differences among HFpEF patients and HC, indicate clear separations between the two groups (R2Y = 0.93, Q2 Y = 0.47, Fig. 1A). The results of the permutation test strongly indicated that the original model was valid (R2 intercept = 0.91, Q2 intercept = − 0.35, Fig. 1B), suggest that the PLS-DA model is not overfitting. The volcano plot revealed a distinct distribution of protein expression changes between the HFpEF and HC groups. We plotted fold changes (using volcano plots) in the levels of identified proteins in HFpEF patients relative to HC groups, A total of 46 proteins showed Significantly differentially expressed proteins (DEPs), among which 36 were significantly upregulated, and 10 were significant downregulated (Fig. 1C, Table S2). of these 21 were associated with inflammation and immune responses, all of that were upregulated in the HFpEF group compared with the HC group. The upregulation of these proteins, particularly IGHV3-64, IGKV2-40, IGLV2-18, and SAA1, suggests an altered immune response in HFpEF patients. The biological function of each DEP was identified through GO analysis and evaluated based on the KEGG. The GO enrichment analysis showed that these DEPs were mainly concentrated in response to stimulus in biological Processes (Fig. 1D), extracellular region in Cellular Component (Fig. 1E), and protein binding in Molecular Function (Fig. 1F). In order to understand the functional characteristics and classification of these DEPs, we conducted pathway enrichment analysis using the KEGG and identified the significant enriched pathways. Based on the KEGG database, the main pathways involved PI3K − Akt signaling and Tuberculosis pathway (Fig. 1G).

### **Metabolomic profiling analysis of HFpEF and HC samples**

**Fig. 2:**
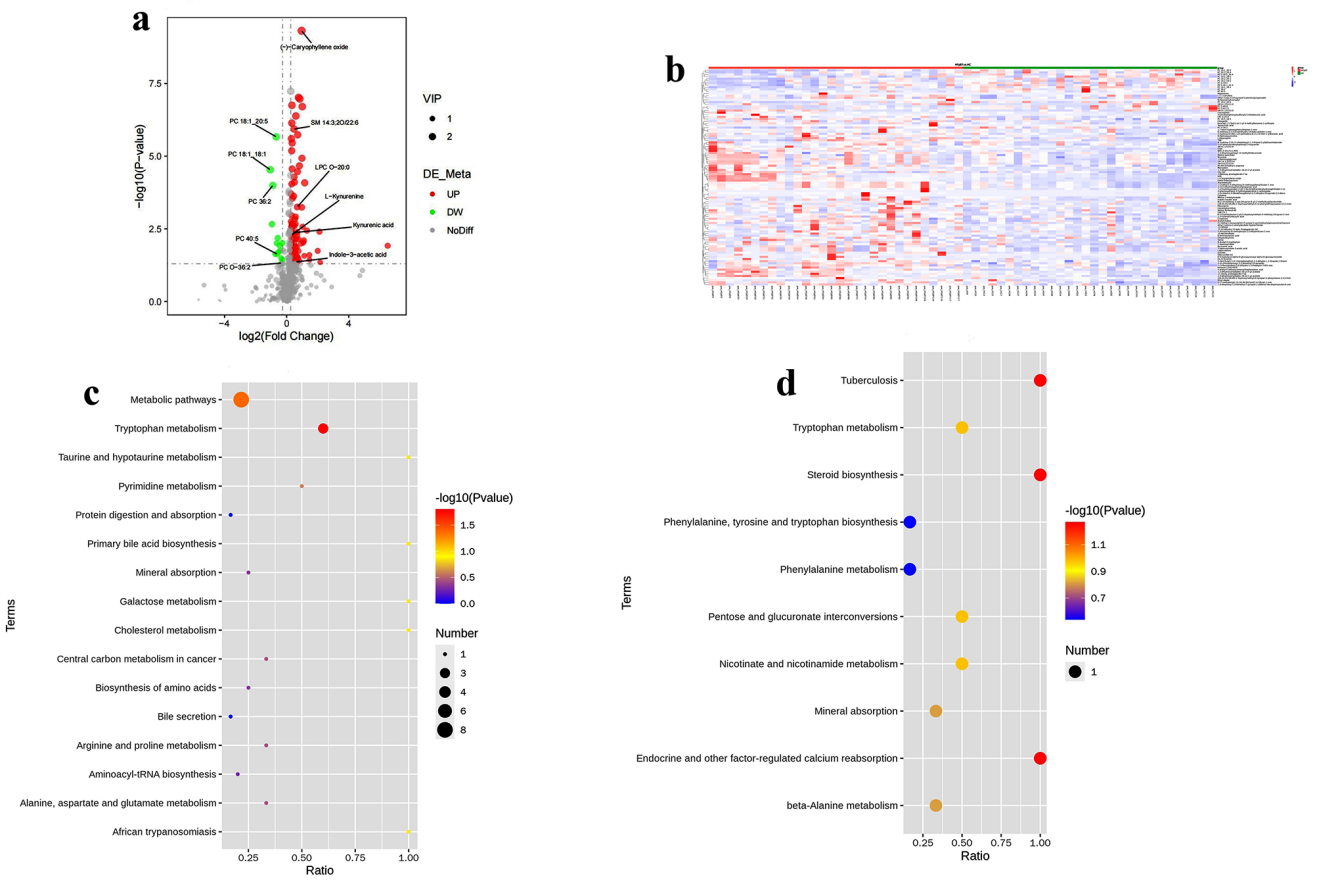
Metabolomics analysis in patients with HFpEF compared to HC. Volcano plot (**A**) and heatmap (**B**) showed differently expressed metabolites in HFpEF compared to HC samples. (**C**, **D**) KEGG enrichment analysis of DEMs in positive ion mode (**C**) and negative ion mode (**D**)

As shown in (Fig.S1A, B), there are distinct clusters of metabolites in HFpEF patients compared with control individuals in both ESI^+^ and ESI^−^. Subsequently, the reliability of the partial least-squares discriminant analysis (PLS-DA) model was assessed using a permutation test. The intercepts of goodness-of-fit (R2) and goodness-of-prediction (Q2) illustrate the PLS-DA model is reliable and not overfitting (Fig. S1C, D). We plotted fold changes (using volcano plots) in the levels of identified metabolites in HFpEF patients relative to HC groups, Totally, 124 DEMs were discovered (Fig. 2A, B). Of these,12 positive downregulated DEMs are phospholipids, which are critical components of all cell membranes and play a key role in lipid metabolism (Table S3). Then, we conducted pathway enrichment analysis using the KEGG and identified the significant enriched pathway in both ESI + and ESI^−^ models. We found that Tryptophan metabolism, Tuberculosis, steroid biosynthesis, Endocrine, and other factor-regulated calcium reabsorption were the top pathways altered in DEMs (Fig. 2C, D).

### Integrated analyses of proteomics and metabolomics data correlation analysis

**Fig. 3:**
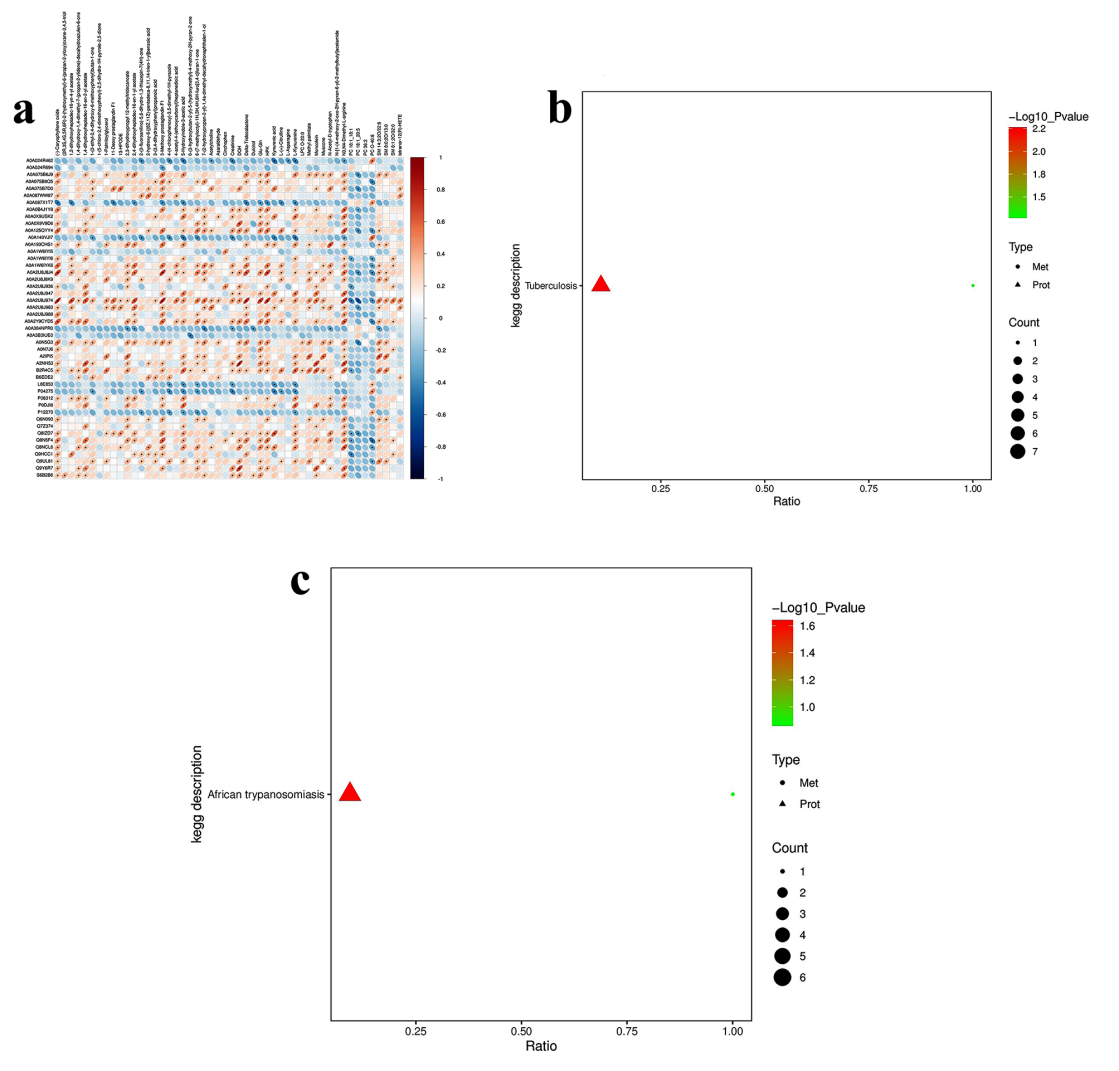
Integrated analysis of proteomics and metabolomics. (**A**) The hierarchical cluster analysis of the DEPs and DEMs. (**B**, **C**) Pathway enrichment analysis of DEPs and DEMs of positive (**B**) and negative (**C**) ion modes

We conducted an integrated analysis of proteomics and metabolomics and found that there were 36 upregulated DEPs, 10 downregulated DEPs, 108 significantly upregulated metabolites, and 16 downregulated metabolites (Fig.S1E); After our initial analysis, we proceeded with a hierarchical cluster analysis between the DEPs and DEMS to appraise protein-metabolites relationship in the HFpEF patients (Fig. 3A). Consequently, a pronounced correlation was observed in the DEPs and DEMs. Integrative analysis via KEGG of the proteins and metabolites showing differential abundance revealed that the main pathways implicated included those associated with Tuberculosis and African trypanosomiasis (Fig. 2B, C). Interestingly, we found that most of the DEPs enriched in this pathway are associated with Inflammation and Immune Response (Fig. 1F, G, Table S4). We reviewed the patients’ history and found that none of the patients with HFpEF had Tuberculosis and African trypanosomiasis. Inflammation and Immune response are thought to play an important role in the pathogenesis of tuberculosis and African trypanosomiasis disease [17, 18]. Therefore, the proteins that may be related to inflammation are enriched in “Tuberculosis and African trypanosomiasis” signaling pathways.

## Discussion

In recent times, proteomics and metabolomics have emerged as innovative tools not only for identifying new risk factors but also for elucidating the underlying mechanisms of cardiovascular disease [9]. Previous studies have revealed aberrant alterations in numerous proteins and metabolites among patients with HFpE [19–22]. For instance, Virginia S. Hahn and colleagues observed decreased levels of fatty acid metabolites in the myocardium of HFpEF patients compared to those with heart failure with reduced ejection fraction (HFrEF). Furthermore, reductions in ketones, metabolites of the tricarboxylic acid cycle, and branched-chain amino acids (BCAA) were noted in HFpEF, indicating a potential inadequacy in utilizing alternative energy sources [23]. Luigi Adamo suggested the feasibility of employing high-content multiplexed proteomics assays in conjunction with the clinical evaluation of LVEF to enhance the identification of clinical phenotypes among HF patients [24]. Nevertheless, a common limitation of these studies lies in their reliance on single-omics technology, which has not provided a comprehensive understanding of the distinctive proteomic and metabolomic patterns in HFpEF compared to those in HC. Given the complexities of biochemical regulation operating across various tiers, deriving meaningful insights from biological data sourced from a single-omics investigation can pose significant challenges. Hence, a holistic approach that integrates diverse omics datasets is instrumental in uncovering the fundamental biological interconnections and enhancing our grasp of the comprehensive biological processes at play in HFpEF.

In this study, we firstly analyzed HFpEF patients and healthy controls plasma samples to evaluate the metabolomics patterns. we found that all of the downregulated DEMs are phospholipids, suggests a potential disruption in cell membrane structure and function, signaling pathways, and inflammatory responses [25]. These disruptions can be due to altered lipid homeostasis, oxidative stress, inflammation, and other metabolic changes [26, 27]. Then, the DEMs between HFpEF patients and HC were used for metabolic pathway analysis. pathways such as Tryptophan metabolism and tuberculosis were significantly altered, suggesting a complex interplay between metabolic disturbances and immune responses in the pathophysiology of HFpEF. It has been reported that inflammatory diseases are associated with dysbiosis- and host-related aberrant tryptophan metabolism [28]. Kynurenine circulates as a metabolite derived from the essential amino acid tryptophan. Masaaki Konishi et al. investigated that Kynurenine was higher in 76 HFpEF patients than in controls. Furthermore, in stable HF patients, an inverse relationship was observed between plasma kynurenine concentrations and both muscle strength and functional capacity, as well as hepatic and renal function [29]. Further research would be necessary to elucidate the exact mechanisms and to determine whether these metabolic changes are a cause or consequence of HFpEF.

Concurrently, an analysis was conducted on patients with HFpEF to discern proteomic variations in comparison to HC. This study identified 36 up-regulated and 10 down-regulated proteins in HFpEF patients compared to HC. We found that most of the DEPs particularly immunoglobulins, suggest an altered immune response in HFpEF patients. The significance of these findings could relate to the pathophysiology of HFpEF, where inflammation and immune responses are increasingly recognized as important factors [30]. The enrichment analysis of the KEGG pathway, focusing on significantly DEPs, has identified several prominent metabolic pathways. Notably, the P13K-Akt signaling pathway is the most significantly enriched pathway associated with HFpEF. Wouter Ouwerkerk’s PhD team identified The PI3K-Akt pathway as a significant association with all-cause mortality in HF [30]. Schulz E et al. have discussed the role of metabolic stress and the JNK pathway, which intersects with the PI3K-Akt pathway, in vascular function and injury [31]. One study also demonstrated that SGLT2 inhibitors touch on their potential effects on cardiac function and HFpEF, which may involve the PI3K-Akt pathway [32]. If SGLT2 inhibitors are found to modulate the PI3K-Akt pathway, either directly or indirectly through their metabolic effects, they might offer therapeutic benefits for patients with HFpEF. This could lead to new therapeutic strategies for HFpEF, a condition that has been challenging to treat effectively.

Subsequently, We next integrated the proteomics and metabolomics datasets to construct a comprehensive plasma profile panorama. Two pathways were significantly enriched in integrated multi-omics, including African trypanosomiasis and Tuberculosis pathway. IgM, IgG1, and Kynurenine which are involved in African trypanosomiasis pathway, IgM and IgG1 were significantly up-regulated in HFpEF compared to HC. Meanwhile, IgG and calcitriol are involved in Tuberculosis pathway, IgG is significantly up-regulated in HFpEF compared to HC. The enrichment analysis that highlighted the Tuberculosis and African trypanosomiasis pathways is particularly intriguing. The up-regulation of IgM and IgG1 in HFpEF patients suggests a heightened adaptive immune response [33]. It is known that chronic inflammation plays a role in the pathophysiology of HFpEF. The kynurenine pathway has also been linked to cardiovascular diseases, as it can lead to the production of metabolites that may have direct cardiovascular effects or modulate immune responses [34]. Calcitriol have been identified as risk factors for cardiovascular disease [35]. The association of calcitriol with HFpEF could reflect alterations in vitamin D metabolism, which has been suggested to be involved in the pathogenesis of heart failure. It has been reported that lower 25-hydroxyvitamin D (25(OH)D) levels were associated with reduced functional capacity in patients with diastolic dysfunction or HFpEF and were significantly predictive of an increased rate of cardiovascular hospitalizations [36].

Understanding the role of immune response and inflammation in HFpEF is crucial, as current treatment options are limited and largely focused on managing symptoms and comorbidities. These insights could lead to new strategies to modulate the immune response or metabolic pathways in HFpEF. It should be underscored that, although the results are indicative, additional investigation is required to confirm these pathways and elucidate their precise functions within the pathophysiological landscape of HFpEF. To the best of our knowledge, this represents the inaugural comprehensive multi-omic investigation that integrates untargeted proteomic and metabolomic datasets from HFpEF patients. It is imperative to acknowledge that the study was a single-center study and involved a limited number of participants. Consequently, to substantiate the findings presented herein, it is essential to undertake additional studies with larger cohorts. Moreover, supplementary experimental work is required to elucidate the mechanisms implicated in the pathogenesis of HFpEF, as suggested by the multi-omic analysis.

## Conclusion

This study identified previously unreported proteomic and metabolomic alterations in HFpEF patients and HC based on the integrative metabolome and proteomics analysis of plasma samples. In conclusion, this study has unveiled a novel approach to elucidating the mechanism of HFpEF, integrating both proteomic and metabolomic analyses into the therapeutic exploration.

### Electronic supplementary material

Below is the link to the electronic supplementary material.

Supplementary Material 1

Supplementary Material 2

Supplementary Material 3

Supplementary Material 4

Supplementary Material 5

Supplementary Material 6

Supplementary Material 7

Supplementary Material 8

Supplementary Material 9

Supplementary Material 10

Supplementary Material 11

Supplementary Material 12

## Data Availability

The sequencing datasets generated and analyzed during the current study are available in the ProteomeXchange Consortium (https://proteomecentral.proteomexchange.org/cgi/GetDataset? ID=PXD053164) via the iProX partner repository with the dataset identifier PXD053164. Other datasets analysed are available in this published article and the Supplementary information files.
