# Supplementary figures and images for: Integrated proteomic and metabolomic profiling reveals novel insights on the inflammation and immune response in HFpEF
Source: BMC Genomics. 2024 Jul 8;25:676. doi: 10.1186/s12864-024-10575-w (PMC11229282; doi:10.1186/s12864-024-10575-w)

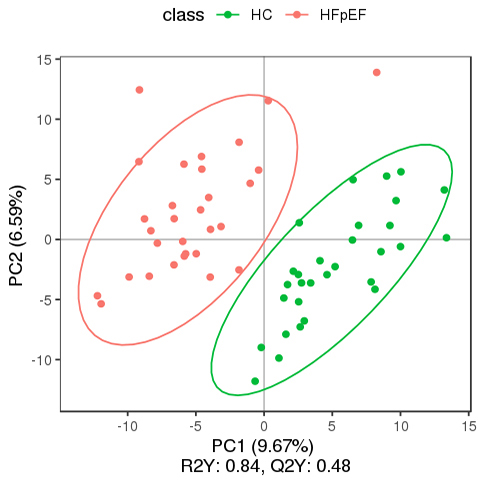

Supplement: Supplementary file 1 — Supplementary Material 1 [file 12864_2024_10575_MOESM1_ESM.jpg]

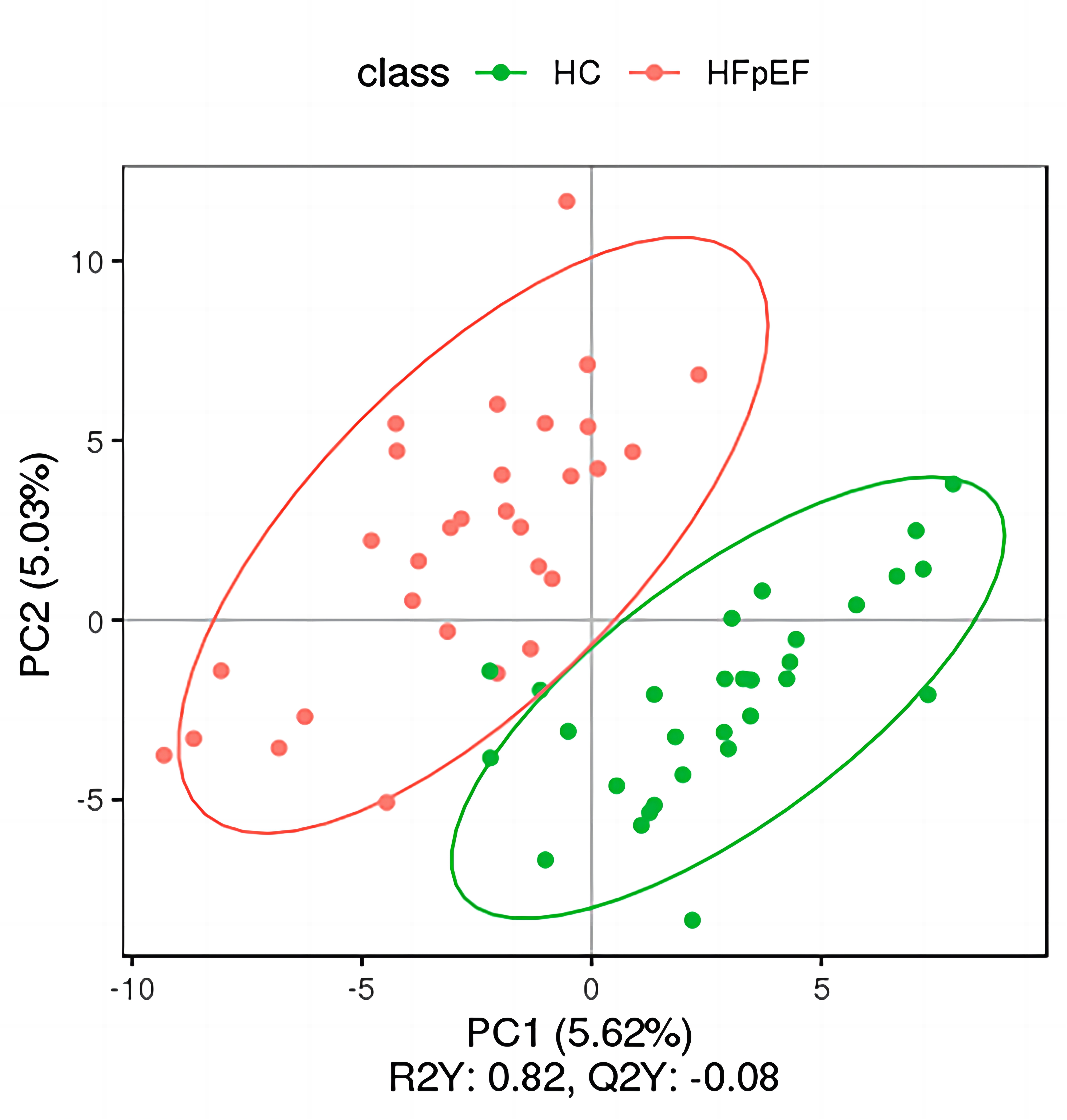

Supplement: Supplementary file 2 — Supplementary Material 2 [file 12864_2024_10575_MOESM2_ESM.jpg]

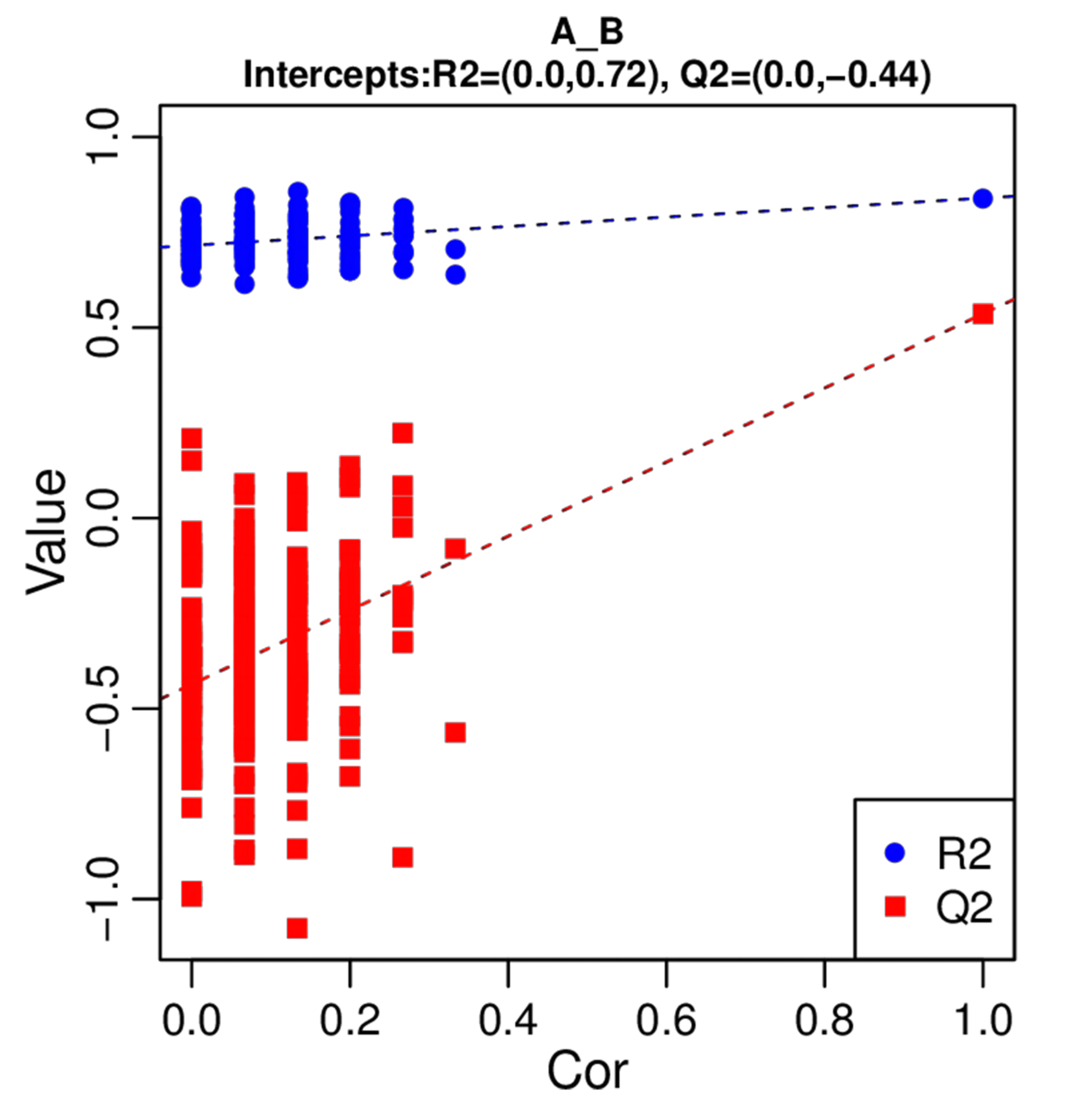

Supplement: Supplementary file 3 — Supplementary Material 3 [file 12864_2024_10575_MOESM3_ESM.jpg]

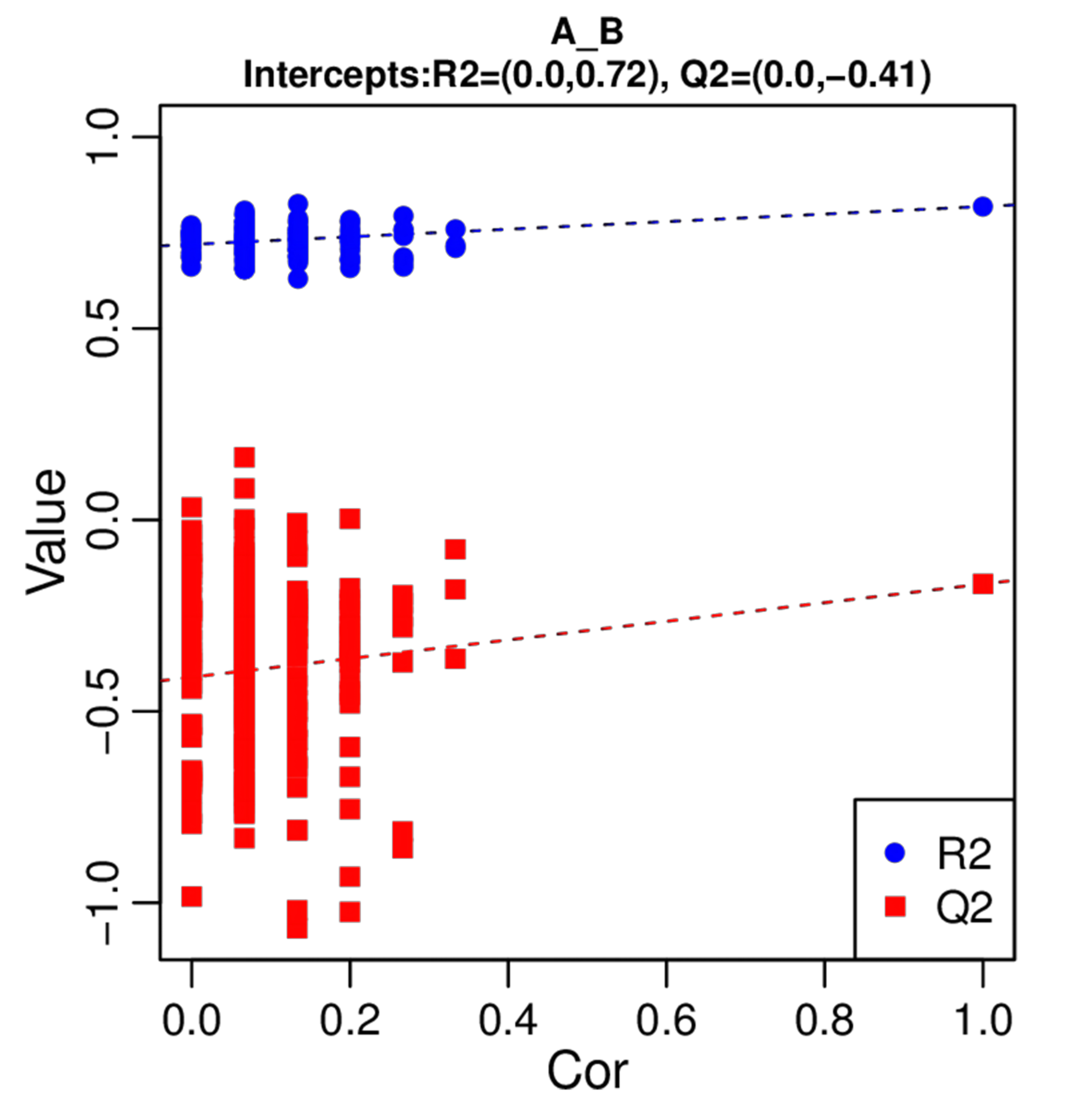

Supplement: Supplementary file 4 — Supplementary Material 4 [file 12864_2024_10575_MOESM4_ESM.jpg]

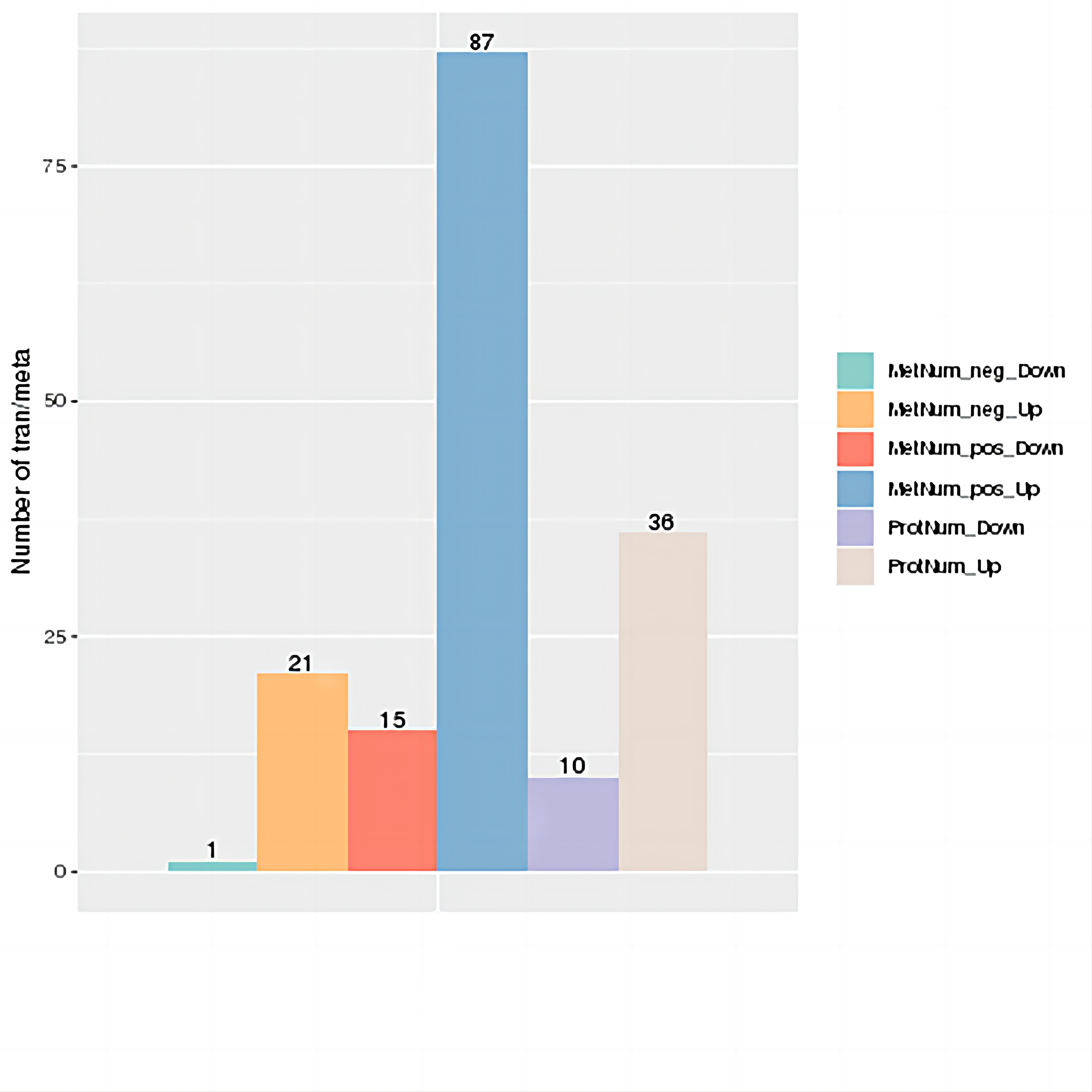

Supplement: Supplementary file 5 — Supplementary Material 5 [file 12864_2024_10575_MOESM5_ESM.jpg]

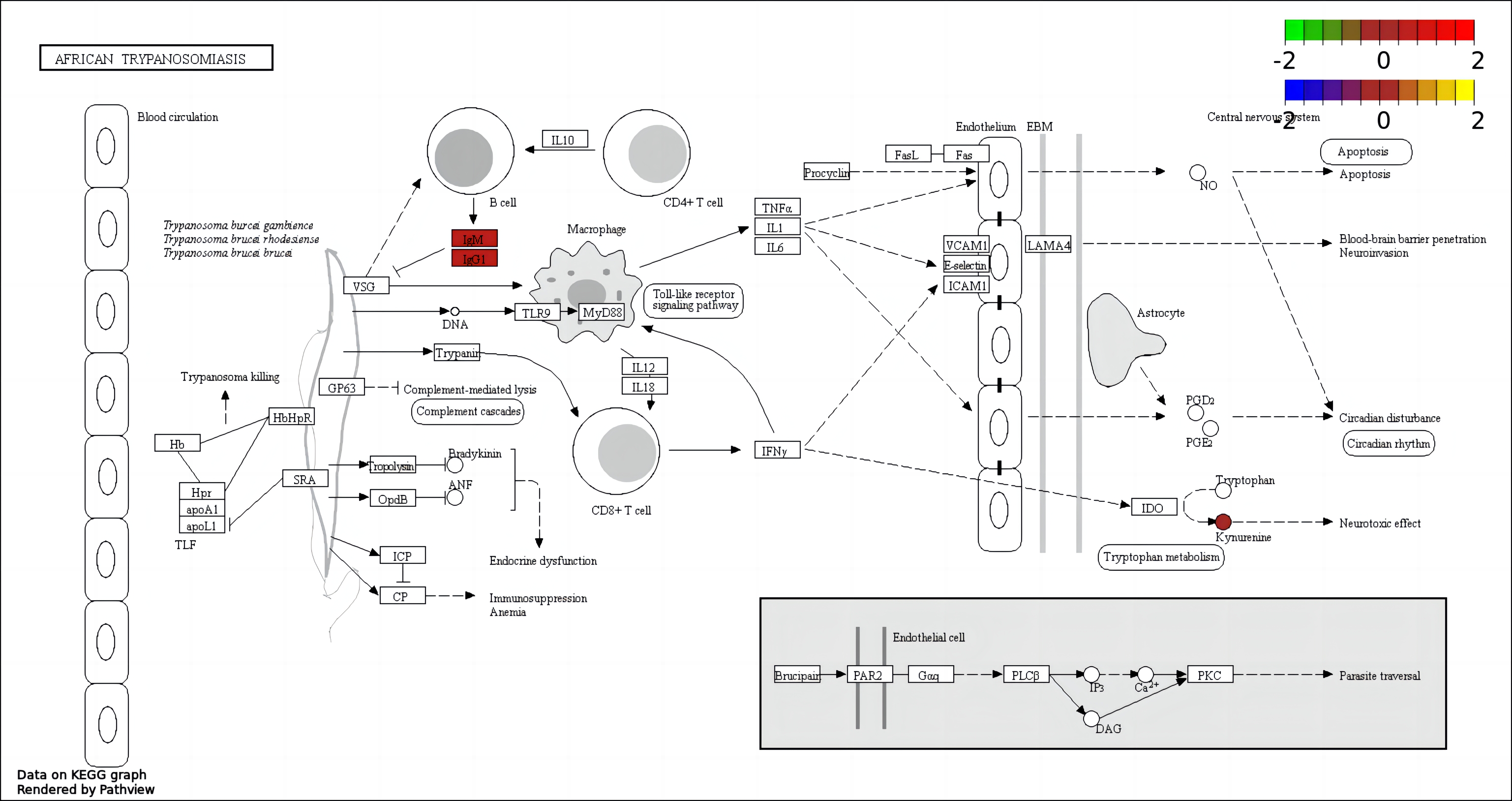

Supplement: Supplementary file 6 — Supplementary Material 6 [file 12864_2024_10575_MOESM6_ESM.jpg]

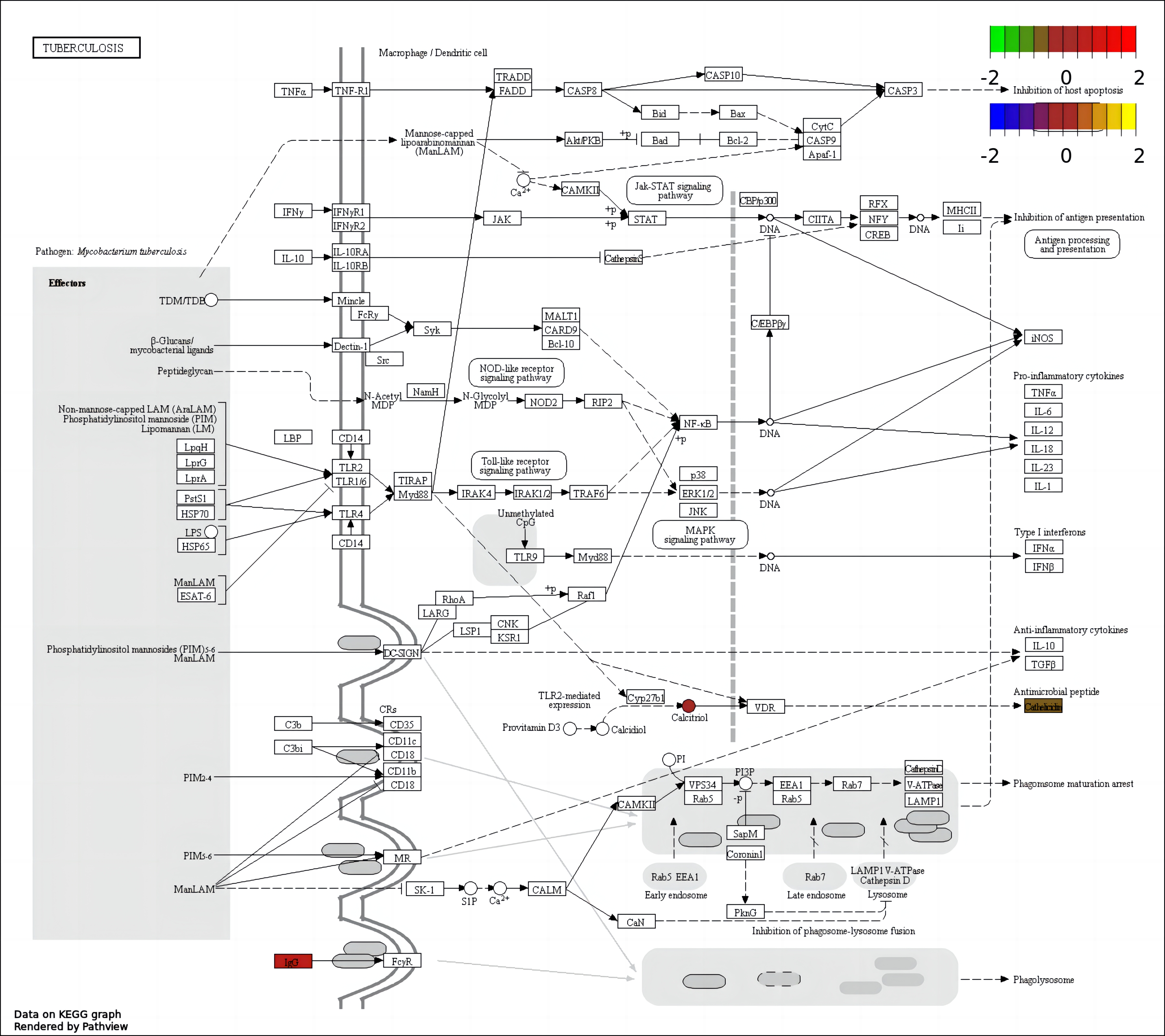

Supplement: Supplementary file 7 — Supplementary Material 7 [file 12864_2024_10575_MOESM7_ESM.jpg]
