# Supplementary material for: Integrated proteomic and metabolomic profiling reveals novel insights on the inflammation and immune response in HFpEF
Source: BMC Genomics. 2024 Jul 8;25:676. doi: 10.1186/s12864-024-10575-w (PMC11229282; doi:10.1186/s12864-024-10575-w)
Supplement: Supplementary file 8 — Supplementary Material 8 [file 12864_2024_10575_MOESM8_ESM.docx]

**SUPPLEMENTARY INFORMATION**

**Methods**

**Proteome Analysis**

**LC-MS/MS Analysis-DIA mode**

Mobile phases A (0.1% FA in H2O) and B (0.1% FA in 80% ACN) were used to develop a gradient elution. A half sample containing 4 μg fraction supernatant and 0.8 μl iRT reagent was injected into the EASY-nLCTM 1200 UHPLC system (Thermo Fisher, Germany) coupled with an Orbitrap Q ExactiveTM HF-X mass spectrometer (Thermo Fisher, Germany) operating in the data-independent acquisition (DIA) mode with spray voltage of 2.1 kV, Nanospray Flex™（ESI）and capillary temperature of 320°C. For DIA acquisition, the m/z range covered from 350 to 1500. MS1 resolution was set to 60000 (at m/z 200), full scan AGC target value was 5×105, the maximum ion injection time was 20 ms. Peptides were fragmented by HCD in MS2, in which resolution was set to 30000 (at 200 m/z), AGC target value was 1×106, a normalized collision energy of 27%. The scan window information was as listed in Table 4 and the raw data of MS detection was named as “.raw”.

**nanoElute- tims TOF pro2**

Prepare mobile phase A (100% water, 0.1% formic acid) and B (80% acetonitrile, 0.1% formic acid). The lyophilized powder was dissolved in 10 μL solution A and centrifuged at 14000 g for 20 min at room temperature. 200 ng supernatant was used for detection. Shotgun proteomics analyses were performed using a nanoElute UHPLC (Bruker, Germany) system coupled with an Tims TOF pro2 (Bruker, Germany) mass spectrometer operating in the data-dependent acquisition (DIA) mode . Peptides were separated in a home-made analytical column (25 cm×75 μm, 1.6 μm), using a linear gradient elution as listed in Table 3. The separated peptides were analyzed by Tims TOF pro2, with ion source of Captive Spray, spray voltage of 1.5 kV. Full scan range was from m/z 100 to 1700, Ramp time was 100 ms and Lock Duty Cycle was 100%. The window size was 25Da, the number of mobility windows is 2. The raw data of MS detection was named as “.d”.

**Data analysis**

**The identification and quantitation of protein**

In order to improve the quality of analysis results, the Spectronaut-Pulsar further filtered the retrieval results: Peptide Spectrum Matches (PSMs) with a credibility of more than 99% was identified PSMs. The identified protein contains at least 1 unique peptide. The identified PSMs and protein were retained and performed with FDR no more than 1.0%. The DIA data was imported into Spectronaut (Biognosys) software to generate a DDA library and ion-pair chromatographic peaks were extracted. Matching the ion and calculating peak area to achieve the qualitative and quantitative of peptides. The iRT was added to the sample for correcting retention time, and the precursor ion Qvalue cutoff was set to 0.01. The protein quantitation results were statistically analyzed by T-test. The proteins whose quantitation significantly different between experimental and control groups, (p < 0.05 and (FC > 1.5 or FC < 0.67 [fold change, FC]), were defined as differentially expressed proteins (DEP).

**The functional analysis of protein and DEP**

Gene Ontology (GO) and InterPro (IPR) functional analysis were conducted using the interproscan program against the non-redundant protein database (including Pfam, PRINTS, ProDom, SMART, ProSite, PANTHER), and the databases of COG (Clusters of Orthologous Groups) and KEGG (Kyoto Encyclopedia of Genes and Genomes) were used to analyze the protein family and pathway. DEPs were used for Volcanic map analysis, cluster heat map analysis and enrichment analysis of GO, IPR and KEGG. The probable protein-protein interactions were predicted using the STRING-db server (<http://string.embl.de/>).

**Metabolome Analysis**

**UHPLC-MS/MS analysis**

UHPLC-MS/MS analyses was conducted using the Vanquish UHPLC system (ThermoFisher, Germany) coupled with an Orbitrap Q Exactive^TM^ HF or Orbitrap Q Exactive^TM^HF-X mass spectrometer (Thermo Fisher, Germany) . Samples were injected onto the Hypersil Gold column (100×2.1 mm, 1.9μm) at a flow rate of 0.2 mL/min over a 12min linear gradient. positive ion mode eluents included 0.1% formic acid in water (eluent A ) and Methanol (eluent B ), while negative ion mode eluents consisted of 5 mM ammonium acetate, (pH 9.0, eluent A) and methanol (eluent B).The elution profile was as follows: 1.5 min with 2%B ;3 min with 2-85% B;10 min with 85-100% B；10 min with 100-2% B and 12 min with 2% B. The Q Exactive^TM^ HF mass spectrometer operated under the following conditions: positive/negative ion mode,3.5 kV spray voltage, 320℃ capillary temperature, 350°C Aux gas heater temperature, 10 L/min aux gas flow rate, 35 psi sheath gas flow rate, and an S-lens RF level of 60.

**Data processing and metabolite identification**

raw data from plasma samples were obtained via UHPLC-MS/MS and processed using Compound Discoverer 3.1 (CD3.1, Thermo Fisher) for peak picking, alignment, and metabolite quantitation .key parameters included a 0.2 min retention time tolerance, 5ppm mass tolerance, a signal/noise ratio of 3, 30% signal intensity tolerance, and a minimum intensity threshold. Subsequently, peak intensities were normalized relative to total spectral intensity. predicting molecular formulas was based on molecular ion peaks, fragment ions, And additive ions. Peak matching against the mzCloud (https://www.mzcloud.org/), mzVault and Mass List database was carried out to obtain relative and accurate qualitative data. Statistical analyses were conducted using R (R-3.4.3), CentOS (CentOS release 6.6), and Python (Python 2.7.6), For normally distributed data, standardization was achieved using the formula: raw quantitation value of the sample / (total metabolite quantitation value of the sample/total QC1 sample metabolite quantitation value). compounds with a CV over 30% in QC samples were excluded, enabling metabolite identification and quantification.

**Data Analysis**

Metabolite annotation in plasma samples utilized the KEGG (https://www.genome.jp/kegg/pathway.html), LIPIDMaps (http://www.lipidmaps.org/) and HMDB (https://hmdb.ca/metabolites) database. Using metaX Partial least squares discriminant analysis (PLS-DA) and principal components analysis (PCA) were conducted. A univariate regression (t-test) determined significant differences (P-value). Metabolites meeting criteria of VIP >1 and P-value< 0.05 and fold change≥2 or FC≤0.5 were classified as differentially expressed. Volcano plots generated by ggplot2 in R, facilitated the selection of metabolites based on log2(FC) and -log10(P-value).
