# Supplementary material for: Integrated proteomic and metabolomic profiling reveals novel insights on the inflammation and immune response in HFpEF
Source: BMC Genomics. 2024 Jul 8;25:676. doi: 10.1186/s12864-024-10575-w (PMC11229282; doi:10.1186/s12864-024-10575-w)
Supplement: Supplementary file 9 — Supplementary Material 9 [file 12864_2024_10575_MOESM9_ESM.docx]

**Table S2**

DEPs between HFpEF patients and healthy controls.

| **Protein** | **Description** | **Gene** | **FC** | **Pvalue** | **trend** |
| --- | --- | --- | --- | --- | --- |
| A0A024R462 | Fibronectin 1, isoform CRA_n | FN1 | 0.53 | 0.05 | down |
| A0A024R694 | Actinin, alpha 1, isoform CRA_a | ACTN1 | 0.54 | 0.01 | down |
| A0A087X1T7 | Aggrecan core protein | ACAN | 0.58 | 0.01 | down |
| A0A140VJI7 | Testicular tissue protein Li 61 |  | 0.63 | 0.02 | down |
| A0A1W6IYI5 | N90-VRC38.08 heavy chain variable region (Fragment) |  | 0.65 | 0.03 | down |
| A0A384NPR0 | Epididymis secretory sperm binding protein |  | 0.62 | 0.00 | down |
| A0A3B3IUE0 | Insulin-like growth factor-binding protein 6 | IGFBP6 | 0.61 | 0.02 | down |
| L8E853 | von Willebrand factor | VWF | 0.47 | 0.05 | down |
| P04275 | von Willebrand factor | VWF | 0.47 | 0.03 | down |
| P12273 | Prolactin-inducible protein | PIP | 0.33 | 0.03 | down |
| A0A075B6J9 | Immunoglobulin lambda variable 2-18 | IGLV2-18 | 1.81 | 0.00 | up |
| A0A075B6Q5 | Immunoglobulin heavy variable 3-64 | IGHV3-64 | 1.52 | 0.00 | up |
| A0A075B7D0 | Immunoglobulin heavy variable 1/OR15-1 (non-functional) (Fragment) | IGHV1OR15-1 | 1.93 | 0.01 | up |
| A0A087WW87 | Immunoglobulin kappa variable 2-40 | IGKV2-40 | 1.54 | 0.01 | up |
| A0A0B4J1Y8 | Immunoglobulin lambda variable 9-49 | IGLV9-49 | 1.84 | 0.02 | up |
| A0A0X9USK2 | MS-A6 heavy chain variable region (Fragment) |  | 1.58 | 0.01 | up |
| A0A0X9V9D6 | IBM-B2 light chain variable region (Fragment) |  | 1.58 | 0.02 | up |
| A0A125QYY4 | GCT-A7 heavy chain variable region (Fragment) |  | 1.55 | 0.00 | up |
| A0A193CHS1 | 10E8 light chain variable region (Fragment) |  | 1.65 | 0.01 | up |
| A0A1W6IYI6 | N90-VRC38.07 heavy chain variable region (Fragment) |  | 1.65 | 0.01 | up |
| A0A1W6IYK6 | N90-VRC38.09 light chain variable region (Fragment) |  | 2.30 | 0.00 | up |
| A0A2U8J8J4 | Ig heavy chain variable region (Fragment) | IgH | 1.92 | 0.00 | up |
| A0A2U8J8K9 | Ig heavy chain variable region (Fragment) | IgH | 1.57 | 0.01 | up |
| A0A2U8J936 | Ig heavy chain variable region (Fragment) | IgH | 1.62 | 0.04 | up |
| A0A2U8J947 | Ig heavy chain variable region (Fragment) | IgH | 1.51 | 0.01 | up |
| A0A2U8J974 | Ig heavy chain variable region (Fragment) | IgH | 3.42 | 0.00 | up |
| A0A2U8J983 | Ig heavy chain variable region (Fragment) | IgH | 1.66 | 0.01 | up |
| A0A2U8J989 | Ig heavy chain variable region (Fragment) | IgH | 1.72 | 0.03 | up |
| A0A2Y9CYD5 | Ig heavy chain variable region (Fragment) | IgH | 1.63 | 0.00 | up |
| A0N5G3 | Rheumatoid factor G9 light chain (Fragment) | V-lambda-3 | 1.77 | 0.00 | up |
| A0N7J6 | REV25-2 (Fragment) |  | 1.62 | 0.01 | up |
| A2IPI5 | HRV Fab 026-VL (Fragment) |  | 2.10 | 0.00 | up |
| A2NH53 | Immunogobulin kappa, VJ region (Fragment) |  | 2.15 | 0.02 | up |
| B2R4C5 | Lysozyme | LYZ | 1.59 | 0.00 | up |
| B6EDE2 | Epididymis luminal protein 180 (Fragment) | HEL180 | 1.82 | 0.01 | up |
| P06312 | Immunoglobulin kappa variable 4-1 | IGKV4-1 | 2.15 | 0.01 | up |
| P0DJI8 | Serum amyloid A-1 protein | SAA1 | 1.94 | 0.04 | up |
| Q6N093 | Uncharacterized protein DKFZp686I04196 (Fragment) | DKFZp686I04196 | 1.61 | 0.01 | up |
| Q7Z374 | Uncharacterized protein DKFZp686C02218 (Fragment) | DKFZp686C02218 | 1.61 | 0.01 | up |
| Q8IZD7 | Anti-thyroglobulin heavy chain variable region (Fragment) |  | 1.59 | 0.00 | up |
| Q8N5F4 | IGL@ protein | IGL@ | 1.78 | 0.00 | up |
| Q8NCL6 | cDNA FLJ90170 fis, clone MAMMA1000370, highly similar to Ig alpha-1 chain C region |  | 1.69 | 0.00 | up |
| Q9HCC1 | Single chain Fv (Fragment) |  | 1.70 | 0.00 | up |
| Q9UL81 | Myosin-reactive immunoglobulin light chain variable region (Fragment) |  | 1.88 | 0.00 | up |
| Q9Y6R7 | IgGFc-binding protein | FCGBP | 1.51 | 0.03 | up |
| S6B2B6 | IgG H chain |  | 1.60 | 0.00 | up |

FC, Fold change; VIP, Variable important in projection.
