# Supplementary material for: Integrated proteomic and metabolomic profiling reveals novel insights on the inflammation and immune response in HFpEF
Source: BMC Genomics. 2024 Jul 8;25:676. doi: 10.1186/s12864-024-10575-w (PMC11229282; doi:10.1186/s12864-024-10575-w)
Supplement: Supplementary file 10 — Supplementary Material 10 [file 12864_2024_10575_MOESM10_ESM.docx]

**Table S3**

**Down-regulated DEMs between HFpEF patients and healthy controls.**

| Metabolite Name | Mode | FC | P value | VIP | trend |
| --- | --- | --- | --- | --- | --- |
| PC 18:1_20:5 | pos | 0.631 | 2.1713 | 2.18 | down |
| PC 18:1_18:1 | pos | 0.479 | 2.982 | 2.3 | down |
| PC 36:2 | pos | 0.539 | 0.0001 | 2.07 | down |
| PC O-40:8 | pos | 0.518 | 0.0021 | 1.53 | down |
| PC 19:2_20:4 | pos | 0.672 | 0.0065 | 1.38 | down |
| PC 20:3_20:4 | pos | 0.829 | 0.0096 | 1.28 | down |
| PC 18:2_20:3 | pos | 0.644 | 0.0098 | 1.32 | down |
| PC O-34:2 | pos | 0.725 | 0.0018 | 1.29 | down |
| PC 19:2_19:2 | pos | 0.752 | 0.0174 | 1.16 | down |
| PE O-18:2_20:4 | pos | 0.701 | 0.0197 | 1.18 | down |
| PC 40:5 | pos | 0.652 | 0.0233 | 1.26 | down |
| PC O-42:11 | pos | 0.598 | 0.0234 | 1.13 | down |
| PC 19:2_18:5 | pos | 0.776 | 0.032 | 1.08 | down |
| PE O-16:1_22:4 | pos | 0.83 | 0.036 | 1.15 | down |
| PC O-36:2 | pos | 0.785 | 0.0476 | 1.48 | down |

POS, Positive; NEG, Negative; FC, Fold change; VIP, Variable important in projection.
