# Supplementary material for: Integrated proteomic and metabolomic profiling reveals novel insights on the inflammation and immune response in HFpEF
Source: BMC Genomics. 2024 Jul 8;25:676. doi: 10.1186/s12864-024-10575-w (PMC11229282; doi:10.1186/s12864-024-10575-w)
Supplement: Supplementary file 11 — Supplementary Material 11 [file 12864_2024_10575_MOESM11_ESM.docx]

TableS4

KEGG pathway analysis of DEMs and DEPs

| **Description** | **Ratio** | **Pvalue** | **Count** | **Type** | **ID** |
| --- | --- | --- | --- | --- | --- |
| Tuberculosis | 1 | 0.05 | 1 | Met | Calcitriol |
| Tuberculosis | 0.11 | 0.01 | 7 | Prot | A0A1W6IYI5, A0A2U8J947, A0A384NPR0, B6EDE2 ,Q7Z374 Q8IZD7, S6B2B6 |
|  |  |  |  |  |  |
| African trypanosomiasis | 1 | 0.14 | 1 | Met | L-Kynurenine |
| African trypanosomiasis | 0.1 | 0.02 | 6 | Prot | A0A1W6IYI5, A0A2U8J947, B6EDE2, Q7Z374, Q8IZD7, S6B2B6 |

Note:

Description: The pathway names of the DEMs / DEPs in the KEGG pathway database.

Ratio:The DEMs /DEPs enriched in this pathway to the number of annotated metabolites or proteins in this pathway.

P value: The p-value for pathway enrichment (protein or metabolites)

Count: The number of metabolites or proteins enriched in the pathway.

Type:Data types, derived from metabolomics or proteomics.

ID: ID of metabolites or proteins enriched in the pathway.
