# Supplementary material for: Integrated proteomic and metabolomic profiling reveals novel insights on the inflammation and immune response in HFpEF
Source: BMC Genomics. 2024 Jul 8;25:676. doi: 10.1186/s12864-024-10575-w (PMC11229282; doi:10.1186/s12864-024-10575-w)
Supplement: Supplementary file 12 — Supplementary Material 12 [file 12864_2024_10575_MOESM12_ESM.doc]

**Table S1**

Basic characteristics of the participants.

| variables | HFpEF(n=30) | HC(n=30) | P value |
| --- | --- | --- | --- |
| Male, n (%) | 17 (56.7%) | 15 (50%) | 0.605 |
| Age(years) | 61.47±11.87 | 52.57±15.34 | 0.490 |
| BMI((kg/m2) | 25.33±4.18 | 24.63±3.52 | 0.500 |
| LVEF, % | 61.01±3.65 | 62.80±2.07 | 0.023 |

BMI：Body Mass Index；LVEF：Left Ventricular Ejection Fractions；
